# Roles of Marine Macroalgae or Seaweeds and Their Bioactive Compounds in Combating Overweight, Obesity and Diabetes: A Comprehensive Review

**DOI:** 10.3390/md21040258

**Published:** 2023-04-21

**Authors:** S’thandiwe Nozibusiso Magwaza, Md. Shahidul Islam

**Affiliations:** Department of Biochemistry, School of Life Sciences, University of KwaZulu-Natal (Westville Campus), Durban 4000, South Africa

**Keywords:** diabetes, marine macroalgae, obesity, seaweeds, type 2 diabetes, bioactive compounds

## Abstract

Obesity and diabetes are matters of serious concern in the health sector due to their rapid increase in prevalence over the last three decades. Obesity is a severe metabolic problem that results in energy imbalance that is persistent over a long period of time, and it is characterized by insulin resistance, suggesting a strong association with type 2 diabetes (T2D). The available therapies for these diseases have side effects and some still need to be approved by the Food and Drug Administration (FDA), and they are expensive for underdeveloped countries. Hence, the need for natural anti-obesity and anti-diabetic drugs has increased in recent years due to their lower costs and having virtually no or negligible side effects. This review thoroughly examined the anti-obesity and anti-diabetic effects of various marine macroalgae or seaweeds and their bioactive compounds in different experimental settings. According to the findings of this review, seaweeds and their bioactive compounds have been shown to have strong potential to alleviate obesity and diabetes in both in vitro and in vivo or animal-model studies. However, the number of clinical trials in this regard is limited. Hence, further studies investigating the effects of marine algal extracts and their bioactive compounds in clinical settings are required for developing anti-obesity and anti-diabetic medicines with better efficacy but lower or no side effects.

## 1. Introduction

**Figure 1:**
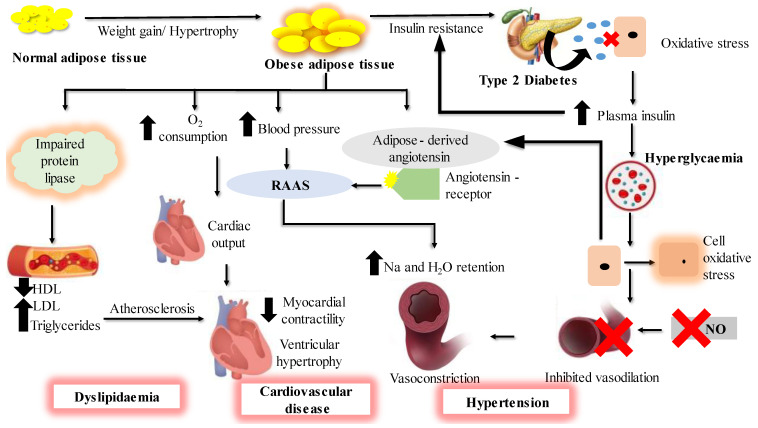
Summary of obesity and diabetes-associated diseases and their interactions. The complications that occur as a result of overaccumulation of fats in the adipose are summarized, which lead to diseases such as T2D, dyslipidemia, cardiovascular disease and hypertension.

Obesity is a metabolic disorder that results from the excessive accumulation of fats in the body [1]. It represents one of the most persistent threats to human health due to a steady increase in its prevalence over the last 30 years, reaching a pandemic level, notably in the developed world and in many developing countries. It is one of the top 10 leading global public health problems [2] and is associated with many metabolic disorders [3] such as type 2 diabetes (T2D), hypertension, heart diseases and dyslipidemia [4], as summarized in Figure 1. These diseases are collectively referred to as metabolic syndrome and they are a matter of serious concern in global public health. Type 2 diabetes is a chronic condition which causes hyperglycemia, insulin resistance and impairment of the way the body responds to the stimuli of insulin. Obesity is usually characterized by increased levels of fasting plasma insulin and abnormally elevated insulin response to an oral glucose load. This suggests that obesity is highly associated with T2D. It has been reported that people with obesity develop insulin resistance, which hinders insulin in performing its cellular actions. Type 2 diabetes is characterized by the inability of insulin to inhibit glucose output from the liver and to promote glucose uptake in adipose and muscle tissues [5,6]; a new term ‘diabesity’ is used to refer to T2D which occurs due to obesity.

**Figure 2:**
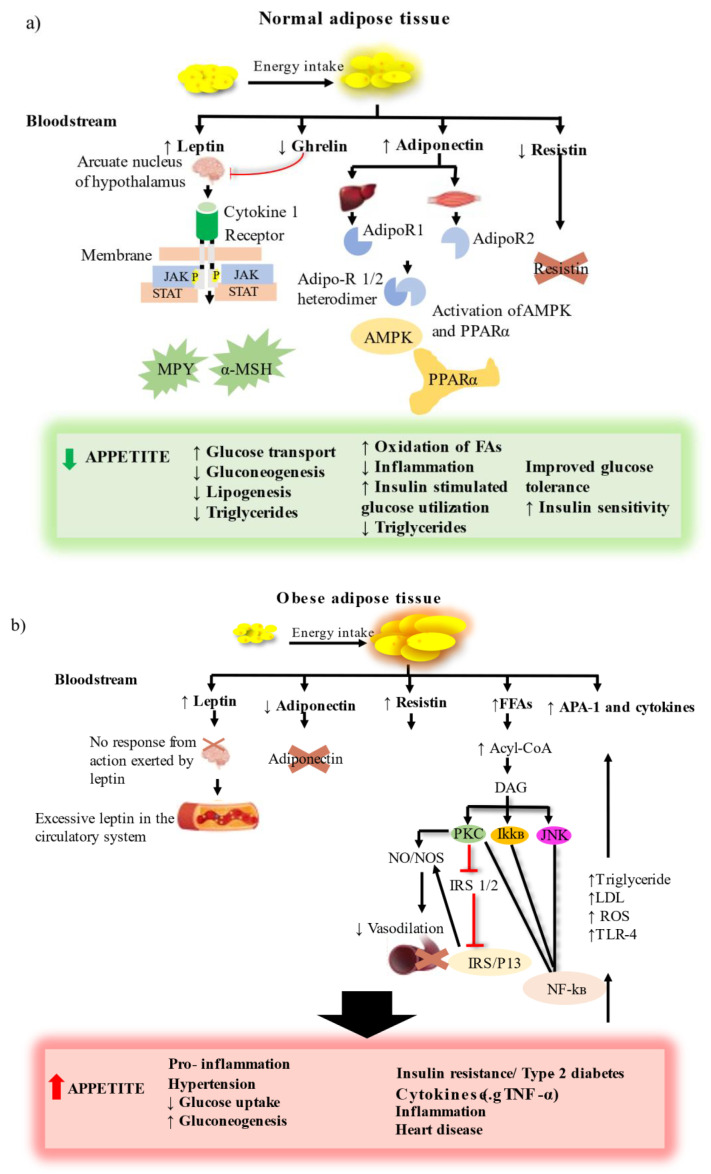
Proposed mechanisms of the adipose tissue secretome (leptin, ghrelin, adiponectin, resistin, FFAs and cytokine factors) in normal (**a**) and obese (**b**) adipose tissues.

Obesity is strongly associated with metabolic abnormalities in adipose tissue, which mainly serves as a fat reservoir. As a result, adipose tissue is regarded as the primary site for the onset and progression of obesity and T2D [7]. Adipose tissue is a complex endocrine organ containing a heterogenous mixture of cells including immune cells, adipocytes and stromovascular cells, and connective tissue matrix and nerve tissue. It is located at different anatomical positions, and the excess (hypertrophy) or deficiency (lipodystrophy) in these adipose depots have harmful metabolic consequences. Hypertrophy of the white adipose tissue (WAT) found in visceral or abdominal positions is associated with adverse effects such as insulin resistance (T2D), dyslipidemia, hyperglycemia, hypertension and inflammation. The adipose tissue ‘secretome’ includes factors such as leptin, cytokines, adiponectin, resistin, complement components, plasminogen activator inhibitor- 1 (PAI- 1) and proteins of the renin–angiotensin system, and their importance in normal and obese adipose tissue is highlighted in Figure 2 [8].

According to World Health Organization (WHO), the worldwide prevalence of obesity is tripled between the years 1975 and 2016 [9]. In 2016, it was estimated that more than 1.6 billion adult people were overweight, of whom 650 million were obese. The prevalence of overweight and obesity broken down by sex showed that 39% of men and 40% of women were overweight, and 11% of men and 15% of women were obese, while over 340 million children and adolescents were obese in the same year. 

On the other hand, diabetes, one of the leading causes of death in the world, affects about 537 million people worldwide [10]. In 2019, it was reported to be in the top nine leading causes of death, accounting for approximately 1.5 million deaths caused directly by diabetes. The prevalence of diabetes increased from 108 million in 1980 to 422 million in 2014 [9], suggesting that the incidence of this disease increased by more than four times during this period. It is anticipated that the rates will continue to rise if no major interventions are made. When these two phenomena of diabetes and obesity are diagnosed in the same person, it is called ‘diabesity’. 

Diabesity is not only a health problem, but also an economic phenomenon. Therefore, it is imperative to study the patterns of diabesity-related expenditures to understand the economic impacts of obesity and diabetes, and to promote the implementation of much needed comprehensive intervention programs and assistance to reverse the diabesity epidemic and promote healthy weight and healthy body mass index (BMI) in all ages. It has been reported that in South Africa, overweight people have higher medical costs compared to people with normal weight [11]. In the majority of cases, the increase in obesity is accompanied by diabetes, suggesting that the observed high cost that comes with obesity should be expected in diabetics. The International Diabetes Federation (IDF) estimated that the global annual healthcare expenditures associated with obesity increased by 316% from the year 2007 ($232 billion) to 2021 ($966 billion) [10]. This shows that obesity and diabetes not only have detrimental effects on human health but also impose a substantial economic burden globally. Moreover, countries with high rates of these diseases must set aside additional budgets to cover their costs. People suffering from either or both of these chronic conditions need to manage them carefully by managing diet, regular physical exercise and pharmacological therapeutics. 

The first line of therapy for obesity and its associated diseases such as T2D is lifestyle changes, which include consuming a healthy and balanced diet, physical activity or exercise, and behavioral changes [12]. However, as much as these non-pharmacological methods are effective, they have low long-term success rates and regaining the lost weight is a major problem. There are pharmacological interventions available; however, these require that they be accompanied with lifestyle changes to facilitate clinically meaningful weight loss. Currently, there are five drugs, namely, orlistat, phentermine/topiramate, lorcaserin and naltrexone/bupropion, that have been approved for the treatment of obesity [13]. Lorcaserin, metformin and DPP- IV inhibitors are used as a treatment for diabetes in overweight patients [14,15,16]. Although a number of pharmacological therapies are available in the market for both diabetes and obesity, none of them are without either short- or long-term side effects. Hence, there has been a growing interest in finding alternative therapies from natural sources which have either no or fewer side effects and which may have better or similar efficacies compared to conventional therapies, with marine algae being one of them.

The inclusion of marine algae as part of a diet is highly recommended because of its nutritional value, being rich in fiber, minerals and polyunsaturated fatty acids [17]. Additionally, they contain bioactive compounds such as fucoxanthin and phlorotannins that are not found in terrestrial plants, which may play a valuable role in modulating chronic diseases. There are several reports showing an association between dietary intake of marine algae and the alleviation of chronic diseases such as hyperlipidemia, cardiovascular disease and cancer [18]. Moreover, all currently available anti-obesity drugs have either short or long-term side effects, and some are still under clinical trial as they still need to be approved by the FDA. Therefore, the demand for anti–obesity drugs derived from nature has increased in recent years due to their lower side effects and costs. 

There are a number of medicinal plants that have been evaluated with the aim to find anti-obesity and anti-diabetic drugs [19,20,21]. However, research on marine algae in this regard is very scanty, although various compounds found in these plants have been proven to have anti-obesity and anti-diabetic effects. There is evidence reporting the potential therapeutic benefits of marine algae and their bioactive compounds in the management of weight and the treatment of diabetes. These attributes have a potential to stimulate interest in the pharmaceutical industries to develop anti-obesity drugs from these compounds. The most recent and advanced in vitro and in vivo studies and clinical trials on bioactive compounds and extracts from marine macroalgae for the treatment and management of obesity and T2D are presented in this review.

## 2. Primary Marine Algal Bioactive Compounds to Treat Obesity and T2D

**Figure 3:**
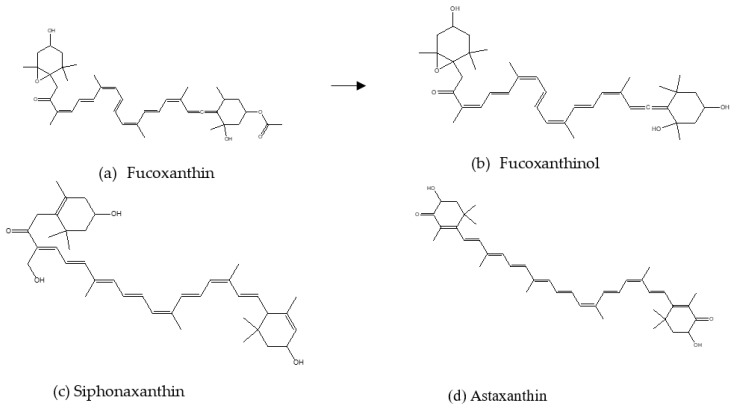
The chemical structures of some major marine algal primary compounds with anti-obesity and anti-diabetic activities.

This section of the review discusses the anti-obesity and anti-diabetic properties demonstrated by primary algal bioactive compounds in vitro, ex vivo, in vivo and human trialss, as shown in Table 1, Table 2 and Table 3, respectively. Figure 3 illustrates some of the major primary compounds in marine algae to depict their functional groups which could be responsible for their anti-obesity and anti-diabetic effects.

### 2.1. In Vitro Studies

Fucoxanthin is an orange-colored carotenoid abundant in edible brown sea algae and diatoms such as *Undaria pinnatifida*, *Laminaria digitata*, *Hijika fusiformis*, *Sargassum fulvellum*, *Laminaria japonica*, *Phaeodactylum tricornutum* and *Isochrysis galbana* [22,23,24]. Fucoxanthin has been shown to have a positive influence on obesity and its comorbidities. A study by Maeda et al. [25] evaluated the anti-obesity effects of fucoxanthin in 3T3-L1 cells and they observed reduced intracellular lipid accumulation and conversion of fucoxanthin to fucoxanthinol, which further suppressed lipid differentiation and accumulation in 3T3-L1 cell lines due to lower glycerol-3-phosphate dehydrogenase activity and downregulated PPARγ. Moreover, fucoxanthinol reduced low-grade inflammation in adipocyte cells [26]. A state of low-grade inflammation is a significant indicator of the development of insulin resistance and advancement to T2D [27]; therefore, fucoxanthin also alleviates T2D. There are only limited studies on the effects of this compound specifically on diabetes; however, since T2D is strongly linked to obesity, the previously mentioned studies also suggest that fucoxanthin ameliorates T2D. The basic chemical structures of fucoxanthin and fucoxanthinol are shown in Figure 3a,b, respectively.

Phlorotannins are phloroglucinol-based polyphenols that are widely distributed in brown algae such as *Eisenia bicyclis*, *Ecklonia cava*, *Ecklonia stolonifera*, *U. pinnatifida*, *Sargassum thunbergia*, *Ishigeo okamurae* and *L. japonica* [28,29]. The ability of phlorotannins to inhibit adipogenesis at different concentrations was evaluated by Jung et al. [30], and they were shown to inhibit lipid accumulation in 3T3-L1 cells. Additionally, the isolated phlorotannins reduced the expression of adipocyte marker genes such as PPARγ and C/EBPα [30]. Moreover, Ko et al. [31] reported not only reduced expression of PPARγ and C/EBPα but also downregulated SREBP1 and FABP4, as well as activation of AMPK which mediates adipogenesis inhibition. In addition, phlorotannins isolated from *E. stolonifera* and *E. bicyclis* showed promising results in controlling postprandial blood glucose by inhibiting PTP-1B and α-glucosidase activities [32]. These results show that phlorotannins have the potential to be used for the treatment of obesity in conjunction with T2D. 

Siphonaxanthin (Figure 3c) is a specific keto-carotenoid found in green algal plants such as *Caulerpa lentillifera*, *Codium fragile* and *Umbraulva japonica* [33]. Li et al. [34] investigated the anti-obesity effects of this compound on 3T3-L1 cell cultures and diabetic KK- Ay mice. It was observed that siphonaxanthin significantly suppressed lipid accumulation and inhibited adipogenesis and protein kinase B phosphorylation in 3T3-L1 cells. Furthermore, the gene expression of C/EBPα, PPARγ, Fabp4 and SCD1 were lowered following the treatment of cells with this compound. Moreover, siphonaxanthin reduced triglyceride accumulation and downregulated fatty acid translocase and Fabp4 [35], which is responsible for fatty acid uptake, by inhibiting hepatic lipogenesis, which is known to contribute to obesity [36]. To the best of our knowledge, the in vitro antioxidant and carbohydrate digestive enzyme potentials of siphonaxanthin have not been explored, although it has demonstrated inhibitory effects on lipogenesis.

Astaxanthin (Figure 3d) is a red-pigmented carotenoid produced by marine animals such salmon, trout, krill, shrimp, crayfish, crustaceans and salmon, as well as algal plants such as *Chlorella zofingiensis*, *Haemotococcus pluvialis*, *Scenedesmus obliqus* and *Chlorococcum* sp. [37,38]. This compound has been reported to be an antioxidant and a potential therapeutic for weight management. Tsai et al. [39] investigated the effects of astaxanthin to inhibit lipid accumulation in 3T3-L1 adipocytes. They found that astaxanthin strongly suppresses lipid accumulation in 3T3-L1 adipocytes in conjunction with the downregulation of genes required for lipogenesis and lipid accumulation in cells. Astaxanthin extracted from *Phaffia rodozyma* showed inhibitory effects on pancreatic lipase, an enzyme responsible for hydrolyzing fats. These inhibitory effects were due to the secondary conformation of lipase by astaxanthin, consequently preventing the binding of lipids on the catalytic site of the lipase [40]. These findings suggest that astaxanthin has the potential to be used as a drug to delay digestion and absorption of fats, thus reducing obesity and T2D. Furthermore, anti-diabetic effects of this compound were demonstrated by Du et al. [41], where it exhibited inhibitory effects on α- glucosidase, an enzyme which catalyzes the hydrolysis of starch into monosaccharides. Therefore, astaxanthin possesses hypoglycemic effects. Astaxanthin has also been reported to be a strong antioxidant [42], which makes this compound an ideal candidate for the treatment of diabetes. 

Neoxanthin, another carotenoid confirmed to have anti-obesity effects, is found in the green leaves of plants and algae. Okada et al. [43] evaluated the ability of neoxanthin to suppress adipocyte differentiation in T3T- L1 cells. It was observed that neoxanthin reduces lipid accumulation and glycerol-3- phosphate dehydrogenase activity. Treatment with neoxanthin also decreased the expression of C/EBPα and PPARγ mRNAs. Moreover, neoxanthin in vegetables showed inhibition of lipid accumulation [44]. There are only limited studies on its in vitro and in vivo anti-obesity and anti-diabetic potential despite it having demonstrated promising results in the aforementioned studies. 

Sulfated fucose (fucoidan) containing polysaccharides are found in brown marine algae such as *Fucus vesiculosus*, *L. japonica* and *U. pinnatifida* [45]. Recently, some interest has been raised concerning its beneficial role in obesity. Kim et al. [46] evaluated the effects of fucoidan on adipocyte differentiation in 3T3 L-1 cells. The results showed lowered adipogenesis in these cells due to the suppression of the expression of genes such as AAP2, ACC and PPARγ. Another study determined the obesity-specific therapeutic action of fucoidan in 3T3-L1 cells and found similar results to the previously mentioned study. In addition, fucoidan decreased the accumulation of lipids and ROS in adipocytes [47]. The antioxidative potential mentioned previously shows that sulfated fucose polysaccharides have anti-diabetic effects. Koh et al. [48] demonstrated the potential of fucoidan extracted from *U. pinnatifida* as an anti-diabetic agent in the treatment and prevention of T2D. It was shown that fucoidan has inhibitory effects on α- glucosidase, α-amylase and amyloglucosidase, while the strongest inhibition was observed in α-glucosidase. Furthermore, the α-glucosidase inhibitory effects of fucoidan extracted from *Sargassum weightii* were reported by Vinoth Kumar et al. [49]. Kim et al. [50] also showed the α- amylase inhibitory effects of fucoidan. In addition, another study suggested that fucoidan can be useful for the treatment and prevention of obesity due to its stimulatory effects on lipolysis [51].

Alginate forms part of the seaweed fibers that are famous for their anti-obesogenic effects. It also forms part of the salts and alginic acid derivatives that are the constituents of brown seaweeds [52] and is dominant in algal species such as *Laminaria* and *Lessonia*. It is mostly used as an emulsification stabilizer in food products by food industries [53], and it has been reported that the addition of this compound in the diet controls the appetite by increasing satiety, reducing food intake and weight reduction [54]. Alginate was evaluated for its pancreatic lipase inhibition potential after cooking and digestion, and the findings showed that this compound maintained its inhibitory effects following being exposed to heat and digestive enzymes [55], thereby suggesting that alginate possesses potential as a therapeutic for obesity. Zhao et al. [56] investigated the antioxidant potential of alginates with different molecular masses isolated from *L. japonica*. The findings showed that alginates with low molecular mass had the highest antioxidant activity on radicals as compared to alginates with high molecular mass, carnosine and ascorbic acid. This antioxidant potential of alginate suggests that it may possess protective effects against the development of diabetes through the inhibition of peroxidation reaction chains. However, to the best of our knowledge, research on alginates in this regard is still very limited.

### 2.2. In Vivo Studies

The preceding section clearly illustrated the in vitro investigation of primary algal compounds as a therapeutic for obesity and T2D. Therefore, it is imperative that the in vivo model studies be highlighted to further demonstrate the potential of these compounds to treat these diseases, as shown in Table 2.

The in vitro studies indicated that fucoxanthin and its metabolite, fucoxanthinol possess potential to prevent obesity and T2D though the inhibition of lipid accumulation, genes responsible for lipogenesis, and carbohydrate digestive enzymes. In this section, we evaluated the benefits of the administration of these compounds in animal models. A study by Grasa-López et al. [57] used a mouse model to investigate the effect of 1 mg/kg oral fucoxanthin on biochemical, physiological and inflammation markers related to obesity and the expression of genes that play major roles in lipid metabolism in white adipose tissue (WAT). It was proven that fucoxanthin administration decreased WAT mass and serum triacylglycerols, improved insulin resistance, reduced blood pressure, decreased serum levels of adiponectin and the expression of leptin, and improved energy expenditure, β-oxidation and adipogenesis by upregulating PPARα, PGC1α, PPARγ and UCP-1.

**Table 1:** Summary of in vitro anti-obesity and anti-diabetic effects of primary algal bioactive compounds and their mechanism of action.

Primary Compounds	Models	Biological Effects	References
Fucoxanthin	3T3-L1 cells	↓ Intracellular lipid accumulation	[25]
		Inhibition of α- amylase	
		Weak inhibition of α- glucosidase	
		↓ Lipid accumulation	
		↓ Glycerol-3-phosphate dehydrogenase activity	
		↓ PPARγ regulation	
Fucoxanthinol	3T3-L1 adipocyte cells and a RAW264.7 macrophage	↓ TNF- α and MCP- 1 mRNA expression	[26]
		↓ protein levels and macrophage cells	
Siphonaxanthin	3T3-L1 preadipocytes	↓ Adipogenesis	[34]
		↓ Adipocyte differentiation	
		↓ Protein kinase b phosphorylation	
		↓ Gene expression of C/EBPα, PPARγ, FABP4 and SCD1	
	3T3-L1 adipocytes	↓ Lipid accumulation	[43]
	HepG2 cell line	↓ Triglyceride accumulation	[35]
		↓ Fatty acid translocase and FABP4 expression	
		↓ Hepatic lipogenesis	
Astaxanthin	3T3-L1 adipocytes	↓ Lipid accumulation	[39]
		↓ Lipogenesis genes	
	Kinetics analysis	Inhibition of pancreatic lipase	[40]
		Inhibition of α- glucosidase	[41]
Neoxanthin	3T3-L1 adipocyte	↓ Glycerol-3- dehydrogenase	[43]
		↓ Expression of C/EBPα and PPARγ mRNA	
		↓ Adipogenesis	
		↓ Lipid accumulation	[44]
Fucoidan	3T3-L1 cells	↓ Expression of AAP2, ACC and PPARγ gene	[46,47]
		↓ Reactive oxygen species (ROS)	
		↓ Lipid accumulation	
		↑ Lipolysis	
		↓ Adipogenesis	[30]
	In vitro without cell line	Inhibition of pancreatic lipase	[31,49,50]
		Inhibition of α- amylase	
		Inhibition of α- glucosidase	
Phlorotannins	3T3-L1 preadipocytes	↓ Lipid accumulation	[30,31]
		↓ Expression of C/EBPα and PPARγ Mrna	
		↓ Expression of SREBP1 and FABP4	
		MAPK activation	
	Carbohydrate digesting enzyme and tyrosine phosphate 1B inhibition	Inhibition of α- glucosidase	[32]
		Inhibition of tyrosine phosphate 1B	
Alginates	Lipid digestive enzyme inhibition, antioxidant activity	Inhibition of pancreatic lipase	[55]
		↓ Reactive oxygen species (ROS)	[56]

Another study showed reduced body weight and white adipose tissue (WAT) size in a high-fat diet (HFD)-induced obesity model of mice after the supplementation of 1.06–2.22% dietary fucoxanthin. Other beneficial effects observed were improved insulin resistance, glucose transporter- 4 (GLUT- 4) mRNA expression, blood glucose and leptin levels. Moreover, there was reduced MCP-1 mRNA expression and increased mRNA expression for β3-adrenergic receptor (Adrb3) [58]. In addition, after the supplementation of 0.2% dietary fucoxanthin, Maeda et al. [59] reported lower inflammatory markers in WAT due to suppressed MCP-1 mRNA expression [59]. These results were further supported by Tan and Hou [60], where they showed the suppression of inflammatory biomarkers such as interleukin-1β (IL-1β), inducible nitric oxide synthase (iNOS), tumor necrosis factor alpha (TNF-α) and cyclooxygenase-2 (COX-2), which was also accompanied by weight loss, reduced inflammation, malondialdehyde (MDA) and myeloperoxidase (MPO) levels after the administration of 0.2–0.6% intragastric fucoxanthin to HFD-induced obese mice [60]. Moreover, fucoxanthin improved lipid and glucose metabolism, reduced blood lipid concentration and promoted glycogen synthesis through gene regulation and IRS-1/PI3K/Akt and AMPK signaling [61]. Further anti-diabetic and anti-obesity potentials of fucoxanthin are highlighted in Table 2. This primary algal compound is an excellent candidate for anti-diabetes and anti-obesity therapeutics due to the previously mentioned results. Its ability to ameliorate both obesity and T2D suggests that it may be used to develop a single drug to treat both obesity and T2D since these diseases usually occur simultaneously. 

The antioxidant and anti-diabetes potential of phlorotannins isolated from *Cystoseira compressa* were evaluated in a study by Gheda et al. [62]. Administration of phlorotannins in diabetic rats resulted in reduced blood glucose, MDA levels and carbohydrate digestive enzyme activities, as well as increased serum insulin and antioxidants. The studies on this compound demonstrate that it has health benefits including ameliorating diabetes and cancer [63]; however, there are no clinical trial studies to further understand the effects of phlorotannins on humans and its bioavailability. 

Oral administration of siphonaxanthin in KK-Ay mice resulted in a significant reduction in the total weight of WAT and lipogenesis, and elevated fatty acid oxidation in adipose tissues [34]. Another study showed that supplementation of diet with siphonaxanthin ameliorated obesity and its effects on HFD-induced obese mice [64].

Astaxanthin showed promising results as an anti-diabetic and anti-obesity agent in the in vitro studies, which stimulated animal model research. Ikeuchi et al. [65] evaluated the effect of 1.2–30 mg/kg bw of astaxanthin administration in HFD-induced obese mice and found that this carotenoid reduces body, liver and WAT weight. Moreover, they observed that astaxanthin reduced liver and plasma triglycerides and total cholesterol [65]. In another study, astaxanthin was proven to improve muscle lipid metabolism during exercise through the inhibitory effects of oxidative Carnitine palmitoyl transferase I (CPT- 1) modification. In addition, fat accumulation was lowered following the administration of this compound at 0.02% dietary concentration [66]. Another study evaluated the effect of astaxanthin on obese insulin-resistant mice and found that this compound has beneficial effects including insulin-sensitivity improvement and prevention of liver damage by lowering CYPE2E1, MPO and nitro-oxidative stress. Moreover, antioxidant status was improved, including reduction of Transforming growth factor- β1 (TGF-β1) expression and lipid deposition [67]. Moreover, 10–40 mg/kg bw of oral astaxanthin ameliorated gestational diabetes and improved the reproduction outcome in pregnant C57BL/KSJ mice [68]. These results demonstrate that astaxanthin has antioxidant potential to alleviate T2D and obesity. 

An in vivo study by Kim et al. [69] found that fucoidan reduces food efficiency, body weight, liver and epididymal fats, plasma triglyceride, total cholesterol, and low-density lipoprotein (LDL) proteins. Moreover, it down-regulated the PPARγ, ACC and adipose-specific FABP- α genes [69]. To support the anti-obesity effects of this compound, another study showed that *Sargassum fusiforme*-derived fucoidan ameliorates insulin resistance and increases antioxidant enzymes in HFD-fed mice [70]. Moreover, the anti-diabetic effects of 45 mg/kg bw of oral fucoidans were shown in C57BL/KSJ *db/db* mice, which showed improved glucose tolerance and insulin sensitivity [71]. 

The above-mentioned in vitro studies showed that alginates possess anti-obesity and anti-diabetes potential. To further support these results, an in vivo study based on HFD-induced obese mice evaluated the effects of 50 mg/kg bw of oral sodium alginate on obesity and related metabolic diseases and its genetic regulation in the colon [72]. The results showed that the sodium alginate alleviated adipose accumulation, inflammation and glucose tolerance. Moreover, this compound resulted in alterations in the colonic genome [72] which were related to the digestion and metabolism of carbohydrates and lipids. These changes in the colon could also be related to those in the previously mentioned study [55], which showed that alginate inhibits a digestive enzyme, pancreatic lipase. Furthermore, unsaturated alginate oligosaccharide showed anti-obesity effects by regulating the disruption of the gut microbiome which resulted from obesity [73]. The anti-diabetes effects of this compound were demonstrated in streptozotocin (STZ)-induced diabetic pigs by regulating blood glucose without the downregulation of the immune system [74].

### 2.3. Clinical Studies

The marine algal primary compounds presented in the in vitro and in vivo studies all demonstrated promising activity to treat obesity and T2D. Therefore, it is essential that their activities be evaluated in humans under clinical interventions for the development of new anti-obesity and anti-diabetic drugs. This section discusses the anti-obesity and anti-diabetic effects of marine algal primary compounds in humans, as shown in Table 3.

Oxidative stress involved in obesity was reported to be significantly lowered in obese individuals following treatment with 5–50 mg of oral astaxanthin [75]. A study by El Khoury et al. [76] reported that individuals whose diet was supplemented with 2.5% alginate had significantly reduced appetite compared to those who did not receive the supplement. The mechanism behind the reduced energy intake following the consumption of alginate includes delayed gastric emptying, increased viscosity of food undergoing digestion and attenuated nutrient absorption in the small intestine upon alginate gel formation [77]. Furthermore, a randomized, double-blind clinical trial investigated the effects of fucoidan administration (500 mg orally) on insulin secretion and insulin sensitivity in obese adults. The results showed a significant decrease in diastolic blood pressure along with increased insulin levels and insulin sensitivity. Reduced LDL-cholesterol levels were also observed following fucoidan administration [78]. Moreover, another study suggested that fucoidan can be useful for the treatment and prevention of obesity due to its stimulatory effects on lipolysis [51].

In a 12-week intervention trial, alginate supplementation in conjunction with energy restriction resulted in more weight loss in comparison with placebo controls in obese individuals [79]. It was shown that the supplementation of human diet with 1.5 g sodium alginate resulted in a lower daily energy intake of the participants compared to controls. Moreover, alginate showed anti-diabetes effects in humans by reducing triglycerides and glucose levels in the body in obese subjects [79]. These results suggested that alginate may be utilized as an additive to weight-reducing diets and in controlling T2D. The results from in vitro, in vivo and clinical trials showed that alginate has anti-obesity and anti-T2D effects; therefore, marine algal-derived drugs can be developed to simultaneously treat these diseases. However, further human trials are still required for other compounds that have only been demonstrated their anti-obesity and anti-diabetic effects in in vitro and in vivo animal models in order to confirm their activities.

**Table 2:** In vivo anti-obesity and anti-diabetic effects of primary marine algal compounds.

Algal Compounds	Dose/Concentration and Route of Administration	Model	Biological Effects	References
Fucoxanthin	Standard dose: 1 mg/kg, intragastric administration	HFD-induced obesity	↓ WAT mass, serum triacylglycerol	[57]
			↓ Expression of leptin and IL-6	
			Upregulation of UCP- 1 expression in BAT, PPARα, PGC1α and PPARγ	
			↑ β- Oxidation, energy expenditure	
			↓ Serum level of adiponectin and leptin expression	
			Improved insulin, reduced blood pressure	
			↓ Adiponectin concentration	
	Low dose 1.06% and high dose 2.22%, dietary supplement	HFD-induced obesity	↓ Weight gain and WAT size	[58]
			↓ Hyperglycemia	
			↓ Hyperinsulinemia	
			↓ Hyperleptinemia	
			↓ Adipose tissue size and weight gain	
			↑ UCP-1 expression	
			↑ GLUT- 4 mRNA expression	
			↑ Adrβ3 mRNA expression	
	0.2% fucoxanthin, dietary supplement	Diabetic/obese KK-Ay mice	Improved insulin, blood glucose and leptin	[59]
			↓ MCP- 1 mRNA expression	
			↑ mRNA expression of Adrβ3	
			↓ Inflammatory markers	
			↓ Lipid accumulation	
			↓ Decreased glycerol-3-phosphate dehydrogenase activity (lipid differentiation)	
			Upregulation of UCP- 1 expression in BAT ↓ PPARα, PGC1α and PPARγ	
	Doses: 0.2–0.6%, intragastric administration	HFD-induced obesity	↓ Weight gain	[60]
			↓ Mammary gland inflammation	
			↓ MDA levels	
			↓ Myeloperoxidase (MPO)	
			↓ Production of IL-1β, TNF-α, iNOS and COX-2	
	0.1%, dietary supplement	Diabetic model KK-*Ay*	Improved glucose tolerance	[75]
			↓ Proinflammation	
	Low dose 0.2% and high dose 0.4%, dietary supplement	C57BL/KsJ- db/*db* mice	Improved insulin resistance	[26,61]
			Improved lipid metabolism	
			↑ Regulation IRS-1/PI3K/AKT and AMPK	
			↓ Plasma lipid levels	
	Standard dose: 0.2%, dietary supplement	Diabetic/obese KK-Ay mice	↑HDL and non-HDL cholesterol	[80]
			↑ SREBP1, SREBP2	
			↓ Food intake	
			↓ Epididymal WAT gain	
	Standard dose: 0.2%, dietary supplement	Diabetic/obese KK-Ay mice	↓ Hyperglycemia	[81]
			↓ Hyperinsulinemia	
			↑ GLUT- 4 mRNA expression	
	Standard dose: 400 mg/kg, intragastric administration	STZ-induced diabetes mice	↓ Hyperinsulinemia	[82]
			↓ Plasma triglyceride	
			↓ LDL cholesterol	
			↑ Regeneration of pancreatic β cells	
Astaxanthin	Low dose 1.2 mg/kg, medium dose 6 mg/kg and high dose 30 mg/kg, dietary supplementation	ddY mice	↓ Body and weight, and WAT size	[65]
			↓ Liver and plasma triglyceride and total cholesterol	
	Standard dose: 0.02%, dietary supplementation	ICR mice	↑ Fat utilization during exercise	[66]
			↑ CPT- 1 activation	
			↓ Fat accumulation	
	Standard dose: 6 mg/kg bw, oral administration	High fat and high fructose diet (HFFD)-fed model	↑ Insulin sensitivity	[67]
			↓ Liver damage by ↓ CYP2E1, myeloperoxidase, nitro-oxide stress	
			Improved antioxidant activity	
			↓ Lipid deposition	
			↓ TGF-β1 expression	
	Standard dose: 30 mg/kg bw, form of administration not indicated	Pregnant C57BL/KsJ db/+	↑ Antioxidant activity	[68]
			Improved glucose tolerance	
			Improved reproductive outcomes	
			↑ Regeneration of pancreatic β cells	
	Low dose10 mg/kg bw, medium dose 25 mg/kg bw, high dose 40 mg/kg bw, oral administration	C57BL/KsJ+/+ (wild-type) and C57 BL/KsJdb/+(db/+)	Improved insulin sensitivity	[83]
			Improved glucose tolerance	
			Improved antioxidant activity	
			Improved reproductive outcomes	
			↑ GLUT- 4 mRNA expression	
			↓ Inflammation	
Fucoidan	20 mg/kg bw, dietary supplementation	Male ICR mice	↓ Fasting blood glucose	[64]
			Restored phosphorylation of Akt	
			↓ Malondialdehyde (MDA)	
			Activated Nrf2 pathway	
			↑ GSH/GSSG ratio	
			↑ Antioxidant enzymes	
	Low dose 1.5 and high dose 2.0%, dietary supplement	C57BL/6 mice	↓ Triglycerides, total cholesterol and LDL proteins	[84]
			↓ Body weight, liver and epididymal fats	
			↓ Food efficiency ration	
	Standard dose: 45 mg/kg bw, oral administration	C57BL/KSJ db/db mice	↓ Hyperglycemia	[71]
			↓ Blood glucose levels	
Alginates	Standard dose: 50 mg/kg, oral gavage	HFD-induced obese mice	↓ Fat, cholesterol and triglyceride accumulation	[72]
			Alteration in colonic genome for immune regulation	
			↓ Blood glucose levels	
	Standard dose: 1 mL/100 mg bw, oral gavage	HFD-induced obese mice	Alteration in colonic genome for immune regulation	[73]
			↓ Inflammatory bacteria	
			Regulate gut microbiota	
			↓ Hyperlipidemia	
			↓ Hyperinsulinemia	
			↓ Blood glucose levels	
	Standard dose: 50 mg/kg, macroencapsulation of islets	STZ-induced diabetes pigs	↓ Blood glucose levels	[74]
			↓ Diabetes for 6 months	
Phlorotannins	60 mg/kg bw, oral gavage	STZ-induced diabetes rat	↓ MDA levels	[62]
			↓ Blood glucose	
			↓ α-glucosidase and α-amylase activities	
			↑ Serum insulin	
			↑ Antioxidant activity	
			↑ Hepatic glutathione and AMPK-α	
Siphonaxanthin	Standard dose: 1.3 mg/kg bw, oral gavage	KK-Ay mice	↓ Total weight of WAT	[34]
			↓ Lipogenesis	
			↑ Fatty acid oxidation in adipose tissues	
	Standard dose: 0.016%, dietary supplement	C57BL/6JhamSlc-Ob/Ob	↓ Plasma glucose and alanine transaminase (ALT)	[64]
			↓ Lipid peroxidation	
			↑ Antioxidant signaling β-Oxidation	

## 3. Secondary Marine Algal Compounds to Treat Obesity and T2D

**Figure 4:**
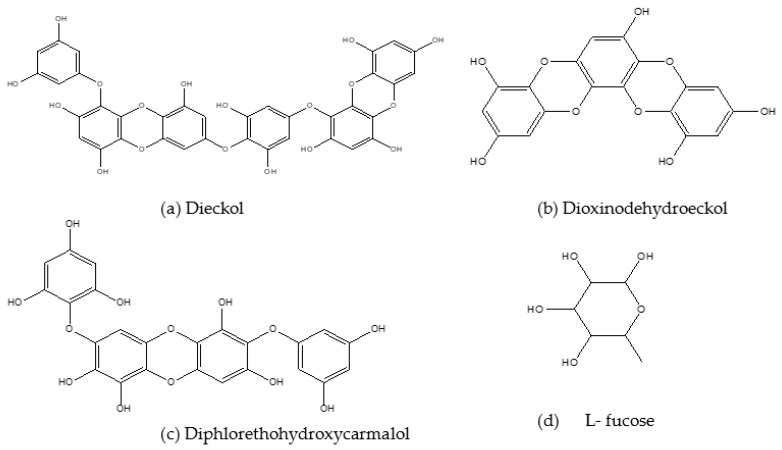
Chemical structures of some major secondary marine algal bioactive compounds with anti-obesity and anti-diabetes activities.

A summary of the anti-obesity and anti-diabetes properties of secondary algal bioactive compounds demonstrated in different in vivo and in vitro model studies are presented in Table 4 and Table 5, respectively. The following section presents the anti-obesity and anti-diabetes effects of secondary bioactive compounds from marine algae. The chemical structures of some major marine algal secondary compounds are shown in Figure 4.

### 3.1. In Vitro Studies

Dieckol (Figure 4a) is a major phlorotannin [85] and in conjunction with seapolynol it has inhibitory effects on both lipid accumulation and 3-hydroxyl-methyl glutaryl coenzyme reductase in 3T3-L1 pre-adipocytes [86]. *E. cava*-isolated dieckol also exerted inhibitory effects on lipid accumulation and genes responsible for adipogenesis in 3T3-L1 cells. There was also decreased expression of C/EBPα, FABP4 and PPARγ mRNA and upregulation of the MAPK pathway. Further anti-obesity effects of this compound were depicted by Jung [87], where dieckol extracted from *E. bicyclis* ethanol extract had strong inhibitory effects on pancreatic lipase enzyme. Moreover, this compound showed higher α-amylase and α-glucosidase inhibition compared to acarbose, suggesting that it possesses both anti-obesity and anti-diabetes potential. 

Other phlorotannins like Dioxinodehydroeckol (DHE) (Figure 4b) and Pyrogallol-Phloroglucinol-6,6′-Bieckol (PPB) that are also derived from *Ecklonia cava* have demonstrated similar effects in alleviating obesity and T2D. Dioxinodehydroeckol was reported to reduce lipid accumulation and downregulate the expression of PPARγ, SREBP1, C/EBP-α and specific adipocyte gene promoters in 3T3-L1 adipocytes [88]. This compound (DHE) also demonstrated the capability to be a candidate for developing drugs and nutraceuticals to delay glucose digestion and absorption by inhibiting α-glucosidase and α-amylase enzymes [89]. Moreover, DHE demonstrated antioxidant activities in a study by Kim et al. [90].

Kang et al. [91] evaluated the inhibitory effects of 6-indole derivatives (indole-2-carboxaldehyde (STC-1), indole-3-carboxaldehyde (STC-2), indole-4-carboxaldehyde (STC-3), indole-5-carboxaldehyde (STC-4), indole-6-carboxaldehyde (STC-5) and indole-7-carboxaldehyde (STC-6)) on adipocyte differentiation in 3T3-L1 adipocytes. They found STC- 1 and STC- 5 to have non-toxic inhibitory effects on adipocyte differentiation, lipid accumulation and the regulation of PPARγ, SREBP1 and C/EBP-α through activation of AMPK- α. Diphlorethohydroxycarmalol (DPHC) (Figure 4c), a compound most abundant in *I. okamurae*, also showed inhibitory effects on lipid accumulation in the study by Kang et al. [91], where they investigated the anti-obesity effects of DPHC in 3T3-L1 cells. The lipid accumulation was inhibited after the treatment with DPHC in a dose-dependent manner, and the expression of adipocyte-specific proteins such as SREBP-1c, PPARγ, C/EBP-α, adiponectin and adipogenic enzymes were also inhibited. Moreover, DPHC inhibited fat accumulation through activation of AMPK and ACC in the adipocytes. This compound also demonstrated anti-diabetes effects by exerting inhibitory effects on α- amylase and α- glucosidase enzymes [91].

### 3.2. In Vivo Studies

In addition to the α-amylase and α-glucosidase inhibitory effects of dieckol presented in the in vitro studies, Lee et al. [92] also observed delayed dietary carbohydrate absorption, which was also accompanied by reduced blood glucose levels, following the treatment of diabetic mice with dieckol. This compound has also been reported to suppress lipid accumulation in adipocytes of mice and zebra fish with the inhibition of adipogenesis. The mechanisms behind these effects include downregulation of adipogenic factors and activation of the AMPK- α pathway [93]. Similar to the study mentioned in the in vitro section above, the administration of dieckol in conjunction with seapolynol to ICR mice fed a HFD resulted in lower body weight gain and a reduction in the levels of total cholesterol, triglycerides and LDL-cholesterol. Moreover, it promoted glucose uptake and increased serum adiponectin levels [86,94].

Pyrogallol-Phloroglucinol-6,6′-Bieckol (PPB) was found to be the most valuable compound among the phlorotannins due to its ability to improve blood circulation and lower blood pressure, lipoproteins and cholesterol upon its administration to diet-induced obese and diet-induced hypertension models of rats [95]. Another study investigated the anti-obesity effects of PPB, which were mediated by lowering the inflammation caused by the receptor for advanced glycation end product (RAGE) and RAGE ligands [96]. Oral administration of this compound resulted in reduced weight in diet-induced obese mice, which was accompanied by adipocyte reduction in size and lower levels of serum triglycerides and cholesterol. Moreover, after the supplementation of PPB, there was a marked inhibition of RAGE ligands, and lowered expression of RAGE, and the binding ratio between the RAGE and RAGE ligands, in the visceral fats of animals. This highlights the anti-obesity effects of PPB mediated by inhibition of RAGE and RAGE ligands and the positive effects of this compound on triglycerides and cholesterol [96]. In addition, PPB restored leptin sensitivity in the brain of leptin-deficient and obese mice [97]. Both DHE and PPB from *Ascophyllum nodosum* were observed to be the polyphenols responsible for reducing DNA damage, reduction of oxidative stress and alleviating obesity [98]. The anti-obesity and anti-diabetes effects of DHE in animal models has not been evaluated despite the results displayed in in vitro studies and clinical trials, as mentioned above.

The anti-obesity effects of indole-3-carbinol indole derivative were investigated in high fat diet-induced obese mice and were found to improve glucose intolerance, increase serum adiponectin concentration, and lower serum glucose, triacylglycerol, insulin and leptin concentrations. The mechanisms behind these effects were the decreased expression of ACC and PPARγ in the adipose tissues of the mice [99]. Xanthigen, an essential source of punicic acid and fucoxanthin, in conjunction with krill oil (KO), were investigated to observe their effects on lipid accumulation in HepG2 liver cells and in HFD-induced obese mice. The results showed that KO reduced lipid accumulation in these cells while xanthigen improved diet-induced hepatic steatosis and reduced bodyweight and adipose tissue mass with no changes in food intake [100]. The anti-obesity mechanisms of xanthigen compound were elucidated in a study by Choi et al. [101], where xanthigen lowered the expression of PPARγ in the adipose tissues of HFD-fed mice. There was also a significant reduction of serum leptin level and expression of HMG-CoA reductase with elevated activation of AMPK α and β and of ACC [101].

In another study, HFD-induced obese mice were orally treated with DPHC. After the treatment with this compound, there was a remarkable reduction in weight gain, triglycerides, LDL-cholesterol, leptin and aspartate transaminase when the level of HDL-cholesterol was increased. In addition, there was downregulation of lipogenic and adipogenic enzymes [102]. A previously mentioned study by Heo et al. [103] showed that DPHC inhibits glucose absorption in diabetic mice.

Monosaccharide L-Fucose (Figure 4d), which can be extracted from the cell walls of *Laminaria* and *Sargassum*, was also proven to reduce weight gain and the expression of adipogenic genes in HFD-induced obese mice. Improved serum adiponectin and glucose and lipid metabolism were reported after supplementation with this compound [104]. There are findings that suggest the use of L- fucose as a compound to treat high fat diet-induced obesity as well as fatty liver. A study by Wu et al. [105] reported that L- fucose administration resulted in decreased body weight, fat accumulation, and hepatic triglyceride elevation in high fat diet-fed mice [105]. The results show the importance and variability of each and every compound that can be utilized in the development of obesity-related treatments.

**Table 3:** Clinical trials on anti-obesity and anti-diabetic effects of primary marine algal bioactive compounds.

Algal Compounds	Dose/Concentration and Route of Administration	Participants	Duration of the Study	Biological Effects	References
Astaxanthin	5 mg and 20 mg, oral dose	*n* = 23 healthy men and women	3 weeks	↓ Lipid peroxidation	[75]
				↑ Antioxidant defense system	
Fucoidan	500 mg, oral dose	*n* = 25 obese and overweight adults	3 months	↓ Diastolic blood pressure	[78]
				↓ LDL cholesterol	
				↑ Insulin secretion and sensitivity	
Alginates	1.25% alginate chocolate milk, 2.5% alginate chocolate or 2.5% alginate solution, dietary supplementation	*n* = 24 healthy men	1 day experiment	↑ Weight loss when supplemented in diet	[76]
				↑ Satiety	
				↓ Energy intake	
				Delayed gastric clearance	
				↑ Viscosity of digesta	
				↓ Nutrient absorption in small intestines	
	1.5 g, dietary supplement	*n* = 96 obese men and women	12 weeks	↑ Weight loss when supplemented in diet	[77]
				↓ Body fat	
				No changes in metabolic risk markers	
	1.5 g, supplemented drink	*n* = 68 healthy men and women	4 weeks	↓ Energy intake	[79]
	1.5 g, supplemented drink	*n* = 14 healthy men	1 day experiment	↓ Cholesterol, triglycerides	[79]
				↓ Blood glucose	

**Table 4:** Summary of in vitro anti-obesity and anti-diabetic effects of secondary algal bioactive compounds and their mechanism of actions.

Compounds	In Vitro Models	Biological Effects	References
Dieckol and seapolynol	3T3 L1 preadipocytes	↓ Lipid accumulation	[86]
		↓ 3- hydroxyl- methyl glutaryl coenzyme reductase	
Dieckol	3T3 L1 adipocytes	↓ Adipogenesis	[31,93]
		↓ Adipogenesis gene and protein expression	
		↑ AMPK pathway	
		↓ Expression of C/EBPα, FABP4 and PPARγ mRNA	
	Lipid digestive enzyme inhibition	↓ Pancreatic lipase	[87]
	Carbohydrate digestive enzyme inhibition	Inhibition of α- amylase	[92]
		Inhibition of α- glucosidase	
Dioxinodehydroeckol (DHE)	3T3 L1 adipocytes	↓ Adipogenesis gene and protein expression	[69]
		↓ Lipid accumulation	
		↑ AMPK pathway	
		↓ Expression of C/EBPα, PPARγ mRNA, SREBP1, FATP1, FAS, LPL, ACS1 and FABP4	
	Carbohydrate digestive enzyme inhibition	↓ α- amylase activity and α- glucosidase activity	[89]
	Antioxidant activity	↓ Antioxidant activities	[90]
Indole derivatives STC-1 and STC- 5/Sargassum thunbergii	3T3-L1 adipocytes	↓ Expression of C/EBPα, SREBP1 and PPARγ mRNA	[91]
		MAPK activation	
Diphlorethohydroxycarmalol (DPHC)	Carbohydrate digestive enzyme inhibition	Inhibition of α- amylase	[103]
		Inhibition of α- glucosidase	

### 3.3. Effective Marine Algae Extracts in the Management of Obesity and T2D

In this section, the most recently studied species of marine algae extracts and their anti-obesity and anti-diabetic effects are presented. Table 6 and Table 7 are summaries of the marine algae species, the dosage or concentration of extracts used and their mode of actions as anti-obesogenic and anti-diabetic agents in in vitro, in vivo and clinical trials, respectively.

The *Sargassum* genus of brown seaweeds are found in the Atlantic, Indian and Pacific oceans, as well as in temperate, subtropical and tropical habitats [106]. This species have numerous pharmacological properties and have been referred as medicinal food due to the pharmacological potential revealed through research. Yende et al. [107] reviewed the potential benefits of different *Sargassum* species. *Sargassum siliquosum* from Australian tropical waters was investigated for its anti-metabolic-syndrome potential using male Wistar rats. *S. siliquosum* extract supplementation to HFD-induced rats with metabolic syndrome resulted in decreased body weight, retroperitoneal fat and liver fat. However, there were no changes or effects on the liver enzyme activities, systolic blood pressure, serum glucose, lipid profile and insulin after 5% dietary supplement in rats [108]. In contrast, Murakami et al. [109] reported that supplementation of 2-6% dietary *S. horneri* to C57BL/6J mice fed a HFD did not reduce weight gain, serum glucose level and insulin resistance. 

Further supporting the potential of *Sargassum* sp. to alleviate metabolic syndrome, extracts of *S. horneri* inhibited the pancreatic lipase in in vitro studies. The 100–300 mg/kg bw of *S. thunbergia* extract treatment significantly reduced bodyweight and fat accumulation in HFD-induced obese mice. In addition, there was reduced serum insulin and triglycerides, liver fats and total cholesterol. Moreover, treatment with *S. thunbergii* ethanol extract resulted in reduced expression of PPARγ and elevated expression of thermogenic genes such as UCP1/3 in the BAT of C57BL/6J mice [110]. Kim et al. [69] investigated the antioxidant activity of *Sargassum miyabei yendo* extract on lipid accumulation and 3T3 L1 pre-adipocyte differentiation. They showed that *S. miyabei yendo* had potent 2,2′-azinobis-3-ehtlbezothiazoline-6-sulfonic acid radical decolorization (ABTS) and 2,2-diphenyl-1-picrylhydrazyl (DPPH) antioxidant activity at IC_50_ values of 0.2868 ± 0.011mg/mL and 0.2941 ± 0.014 mg/mL, respectively. Also, there was markedly reduced lipid accumulation and 3T3 L1 pre-adipocyte differentiation. Furthermore, there was downregulation of expression of C/EBPα, PPARγ mRNA, SREBP1, FATP1, FAS, LPL, ACS1 and FABP4 [69].

*Gelidium* is a red alga, usually used for the extraction of agar [111], and research has shown that it has many biological actions. The anti-obesity effect of *Gelidium elegans* was evaluated [112,113] in an HFD mice model and they found that the supplementation of a 0.5% dietary dose to C57BL/6J ob/obe mice, or 50–200 mg/kg bw to male ICR mice, of this extract reduced weight gain, and subcutaneous and abdominal fat, as well as the inhibition of adipogenesis. Moreover, the blood glucose level was reduced following the administration of the *G. elegans* extract [113]. Kim et al. [114] then evaluated the mechanism behind the weight loss, whether it should be attributed to the inhibition of adipogenesis or not, through a randomized study of obese and overweight individuals. The results of the study showed that there was a significant reduction in weight gain compared to the triglyceride levels following the intake of *G. elegans* extract for 12 weeks. It was therefore concluded that *G. elegans* reduces body weight in overweight or obese patients. *Gelidium amansii*, an edible red alga found in large quantities in Japan, northeast Taiwan and Korea, has been reported to ameliorate obesity and related diseases. After the administration of 0.2–2% of dietary *G. amansii* ethanol extract to HFD-fed mice, Kang et al. [115] found that there was reduced body weight, adipose tissue and liver fat mass, with higher plasma leptin levels compared to the group that was not treated with the extract. Additionally, the mice that were treated with the extract had higher adiponectin levels and reduced expression of adipogenic proteins. Moreover, the expression levels of lipase, AMPK and lipolysis-associated proteins were increased in the extract-treated group [115].

**Table 5:** Summary of in vivo anti-obesity and anti-diabetic effects of secondary algal bioactive compounds and their mechanism of actions.

Algal Compounds	Dose/Concentration and Route of Administration	Models	Biological Effects	References
Dieckol	Low dose 15 mg/kg and high dose 60 mg/kg, dietary supplement.	ICR mice	↓ Lipid accumulation and adipogenesis gene expression	[93]
	Standard dose: 100 mg/kg bw, oral gavage	ICR mice	↓ Blood glucose	[92]
			Delayed carbohydrate absorption	
Dieckol and Seapolynol	Low dose 30 mg/kg and high dose 120 mg/kg bw, oral gavage	C57BL/KsJ-db/db mice	↓ Body weight and water intake	[94]
			↓ Fasting blood glucose and serum insulin levels	
			↓ Cholesterol and triglyceride levels	
			↑ Serum adiponectin, glucose and lipid metabolism	
	12.5-5 mg seapolynol and 0.5- 2 mg dieckol, oral gavage	Male ICR mice	↓ Body weight gain	[86]
			↓ Levels of total cholesterol	
			↓ Triglycerides	
			↓ LDL cholesterol levels	
Pyrogallol-Phloroglucinol-6,6′-Bieckol (PPB)/Ecklonia cava	2.5 mg/kg of PPB, oral gavage	C57BL/6N mice	↓ Body weight	[96]
			↓ Visceral fat/adipocyte size	
			↓ Serum triglycerides	
			↓ Cholesterol	
			↓ Production of RAGE ligands in adipose tissues	
			↓ TNF-a mRNA expression	
	Standard dose: 2.5 mg/kg/day, oral gavage	C57BL/6N mice	Improved blood circulation	[95]
	Standard dose: 2.5 mg/kg/day, oral gavage	C57BL/6N and leptin-deficient (ob/ob) mice	Restored brain leptin sensitivity	[97]
			↑ Macrophage markers and proinflammatory cytokines	
			↑ TLR4 and NF-κB expression	
Indole derivative (indole-3- carbinol)	Standard dose: 5 mg/kg bw, intraperitoneally	C57BL/6J mice	Improved glucose tolerance	[99]
			↑ Serum adiponectin	
			↓ Serum glucose, triacylglycerol, insulin, leptin	
			↓ Expression of PPARγ and ACC	
Krill oil and Xanthigen	Standard dose: 25 g, dietary supplement	C57BL/6J mice	↓ Triacylglycerol accumulation	[100]
			↓ Body weight, adipose mass	
			Improved diet-induced hepatic steatosis	
Xanthigen	Standard dose: 1%, dietary supplement	C57BL/6J mice	↓ PPARγ	[101]
			Activation of AMPK- α and β, and ACC	
			↓ Expression of HMG-CoA reductase	
Diphlorethohydroxycarmalol (DPHC)	Standard dose: 100 mg/kg bw, oral gavage	Male ICR mice	↓ Blood glucose	[103]
			Inhibition of α- amylase	
			Inhibition of α- glucosidase	
	Low dose 25 mg/kg bw and high dose 50 mg/kg bw, oral gavage	C57BL/6J mice	↓ SREBP-1c, FABP4, PPARγ, C/EBP and FAS	[102]
			↓ Levels of triglycerides and low-density lipoprotein cholesterol	
			↓ Leptin and aspartate transaminase	
L-fucose	0.03 g/kg bw, administered intragastrical	C57BL/6 mice	↓ Weight gain and lipid accumulation	[105]
			↓ Hepatic triglyceride elevation	

**Table 6:** Summary of in vitro anti-obesity and anti-diabetic effects of marine algal extracts and their mechanism of action.

Genus	Species Names	Dosages	Models	Biological Effects	References
*Sargassum*	*S. miyabei yendo*	Standard dose: 20 mg/mL	3T3-L1 adipocytes	Potent 2,2′-azinobis-3-ehtlbezothiazoline-6-sulfonic acid radical decolorization (ABTS) and 2,2-diphenyl-1-picrylhydrazyl (DPPH) antioxidant activity (IC_50_: 0.2868 ± 0.011 mg/mL and 0.2941 ± 0.014 mg/mL)	[69]
				↓ Expression of C/EBPα, PPARγ mRNA, SREBP1, FATP1, FAS, LPL, ACS1 and FABP4	
*Gelidium*	*G. amansii*	Low dose 10, medium dose 20 and high dose 40 μg/mL	3T3 L1 adipocytes	↓ PPARγ and aP2 (adipocyte protein 2)	[116]
				↓ ROS-generator, NOX4	
				↑ Adiponectin and GLUT4	
*Ecklonia*	*E. stolonifera*	Dose range: 12–200 µg/mL	3T3-L1 preadipocytes	↓ Lipid accumulation, adipogenesis, adipocyte size	[117,118]
				↑ Lipolysis and browning of WAT	
				↑ MAPK, expression of lipolytic enzymes including ATGL, p-HSL and MGL.	

				↑ Thermogenic genes, CPT1, PRDM16 and UCP-1	
	*E. cava*	Dose range: 12.5–200 µg/mL	3T3-L1 adipocytes	↓ Expression of C/EBPα, SREBP1, FAS, LPL and FABP4	[119]
*Gracilaria*	*G. verrucosa*	Dose range: 1–40 mg/mL	3T3-L1 adipocyte	↓ Lipid accumulation and ROS production	[120]
				Improved glucose uptake	

**Table 7:** Summary of in vivo anti-obesity and anti-diabetic effects of marine algal extracts and their mechanism of action.

In Vivo Studies					
Genus	Species	Dose/Concentration and Route of Administration	Models	Biological Effects	References
*Sargassum* sp.	*S. siliquosum*	Standard dose: 5%, dietary supplement	Male Wistar rats	Decreased body weight, retroperitoneal fat and liver fat	[108]
				No changes in liver enzyme activities, systolic blood pressure, serum glucose, lipid profile and insulin metabolism	
	*S. horneri*	Low dose 2% and high dose 6%, dietary supplement	C57BL/6J mice	↓ Weight gain	[109]
				Improved insulin resistance	
				Inhibited pancreatic lipase	
	*S. thunbergii*	Low dose 100 mg/kg bw, high dose 300 mg/kg bw, dietary	Male C57BL/6 mice	↓ Body weight and fat accumulation	[110]
				↓ Serum insulin and triglycerides, liver fats and total cholesterol	
				↓ PPARγ	
				↑ UCP1 and 3	
*Gelidium* sp.	*G. elegans*	Standard dose: 0.5%, dietary supplement	C57BL/6J-ob/ob mice	↓ Body weight and fat accumulation	[112]
		Low dose 50 mg/kg bw and high dose 200 mg/kg bw, oral gavage	Male ICR mice	Improved insulin resistance	[113]
				↓ Blood glucose	
	*G. amansii*	Low dose 0.5 %, medium dose 1.0% and high dose 2.0 %, dietary supplement	C57BL/6J mice	↓ Weight, adipose tissues and liver fat mass	[115]
				↑ Plasma leptin, adiponectin levels	
				↓ Expression of C/EBPα, PPARγ mRNA, SREBP1, FATP1, FAS, LPL, ACS1 and FABP4	
		Standard dose: 3%, dietary supplement	Hamsters	↓ Expression of C/EBPα, PPARγ mRNA, SREBP1, FATP1, FAS, LPL, ACS1 and FABP4	[121]
				↓ Plasma and liver triglycerides and total cholesterol	
		Standard dose: 5%, dietary supplement	Sprague Dawley rats	↓ Plasma glucose	[122]
				↓ Plasma and liver triglycerides and total cholesterol	
				↓ Plasma adipokines	
*Ecklonia* sp.	*E. stolonifera*	Low dose 50 mg/kg bw, high dose 150 mg/kg bw, oral gavage	Male Institute of Cancer Research (ICR) mice/Male C57BL/6 mice	↓ Serum concentrations of triglycerides, total cholesterol, and LDL	[117,118]
				↑ HDL	
				Body mass	
				Improved insulin resistance and regulation of blood glucose	
				↓ Muscle loss, ↑ expression of MRFs	
	*E. cava*	200 mg/kg bw, oral intubation	C57BL/6 mice	↓ Body weight and hyperglycemia	[123]
				↓ Lipid accumulation, ALT, cholesterol and adiposity	
				↑ mRNA expression of adipogenesis-related genes in adipose tissue	
				Improved insulin resistance	
		Low dose 5 mg/kg, medium dose 25 mg/kg and high dose 150 mg/kg, dietary supplement	C57BL/6 mouse	↓ Body weight	[124]
				↓ Triglycerides, HDL, GOT, GPT	
				↓ Expression of C/EBPα, SREBP1, FAS, LPL and FABP4	
*Gracilaria* sp.	*G. birdiae*	Standard dose: 6 mg/kg body weight, oral gavage	male Mus musculus mice	Reduced weight	[125]
				↓ CCI4-induced damage	
**Clinical Trials**					
**Genus**	**Species**	**Dose/Concentration and Route of Administration**	**Participants**	**Biological Effects**	**References**
*Gelidium* sp.	*Gelidium elegans*	1000 mg/day, orally	*n* = 109 healthy adult volunteers	Waist circumference and hip circumference decreased	[114]
				↓ Fasting glucose, fasting insulin, triglyceride levels	

Similar results to those of the group that was supplemented with the extract were obtained in a comparative study of the anti-obesity effects of a *G. amansii* hot extract and guar gum in HFD-induced obese hamsters [121]. An in vitro study showed that *G. amansii* prevents lipid accumulation by inhibiting the expression of adipogenic factors and reducing ROS production through the regulation of antioxidant and oxidant enzymes during adipocyte differentiation in 3T3-L1 cells [116]. Another study suggested that a 5% dietary dose of *G. amansii* to STZ-induced diabetic rats inhibited lipid accumulation and regulated blood glucose and lipid levels [122].

*Ecklonia*, from the family of *Laminariaceae* and composed of brown seaweeds, has also been reported to be of therapeutic value due to the abundance of bioactive substances found in it. *Ecklonia stolonifera* is an edible brown seaweed that is rich in polyphenolic compounds such as dieckol and phlorotannins. To examine its ability to inhibit lipid accumulation and to stimulate the conversion of WAT to BAT, Jin et al. [117] performed an in vivo animal-model study using HFD-induced obese mice and in vitro studies using 3T3-L1 cells. Animals that were orally administered with 50–150 mg/kg bw of *E. stolonifera* ethanol extract exhibited smaller WAT deposits in the different organ tissues (kidney, liver, lung and spleen) in comparison with the HFD-induced obese animals that were not treated. The extract did not only reduce the WAT mass gain, but also resulted in lower serum concentrations of triglycerides, total cholesterol and LDL-cholesterol, and higher concentrations of HDL-cholesterol. The 3T3-L1 cells of the HFD-induced obese mice treated with the *E. stolonifera* extract exhibited an inhibition of lipid accumulation, lipogenesis and adipogenesis, while lipolysis and conversion of WAT to BAT was promoted [117]. Furthermore, based on the results of the above-mentioned study, Jin et al. [118] then evaluated the biological effects of *E. stolonifera* ethanol extract on obesity-induced hyperglycemia and skeletal muscle loss in HFD-fed obese mice. 

Similar to the results of the previous study, the extract reduced the body mass, lipid accumulation and adipocyte size, while promoting the browning of WAT. Moreover, the *E. stolonifera* extract regulated the plasma glucose and improved glucose tolerance, and it suppressed the loss of skeletal muscle and promoted the expression of myogenic regulatory factors [118]. These findings suggest that the *E. stolonifera* extract prevents skeletal muscle loss resulting from obesity and hyperglycemia. Following the discovery that *Ecklonia cava* has beneficial effects on a type 1 animal model of diabetes [126], Park et al. [123] evaluated the anti-obesity effects of 200 mg/kd bw of oral *E. cava* ethyl acetate extract in HFD-induced obese mice. The extract reduced the bodyweight gain and fats, and hyperglycemia, and it improved insulin sensitivity compared to the HFD-induced obese group that was not treated with the extract. The treated group also exhibited reduced adiposity, alanine aminotransferase (ALT), cholesterol and lipid accumulation. Moreover, another study investigated the inhibitory effects of *E. cava* treatment on 3T3-L1 cell differentiation, and the expression of genes associated with adipogenesis. The results exhibited lower levels of C/EBPα, SREBP-1c, A-FABP and FAS following the treatment with extract [119]. Following these results showing that the extract of this seaweed also prevents obesity at a cellular level, Kim et al. [124] then evaluated the anti-obesity effects of 5–150 mg/kg of dietary *E. cava* enzyme-treated extract on HFD-induced obese mice. Supplementation with the *E. cava* extract resulted in reduced body weight, serum triglycerides, glutamic oxaloacetic transaminase (GOT) and glutamic pyruvic transaminase (GPT) and expression of proteins associated with adipogenesis. 

Some other algal extracts that have been evaluated including *Gracilaria birdiae*, which was shown to have protective properties against carbon tetrachloride (CCI_4_)-induced liver damage in conjunction with decreased bodyweight gain in Mus musculus mice [125]. From the same genus, *Gracilaria verrucosa* was reported to inhibit lipid accumulation and ROS production and improve glucose uptake in 3T3-L1 cells [120]. Another study showed the antioxidant and hypoglycemic potential of *Gracilaria edulis* by using in vitro experimental models [127]. These findings indicate that seaweeds have anti-obesity and anti-diabetes effects, which are attributed to their bioactive compounds, as has been demonstrated in this review.

## 4. Materials and Methods

**Figure 5:**
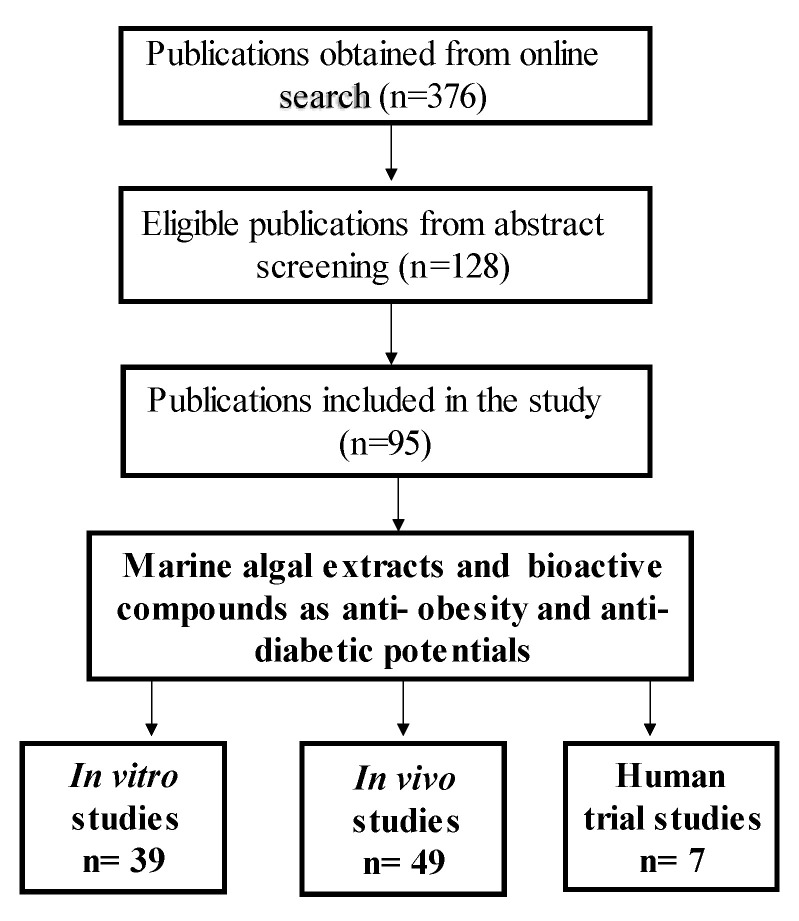
Flow diagram depicting publication selection procedure. The number of publications for each stage of article selection is depicted. The number of articles was refined from 376 to 95 and the selection was based on their relevance to the study.

Online search engines such as ‘Google Scholar’, ‘PubMed’, ‘Scopus’ and ‘ScienceDirect’ were used to search for the literature included in this study. The purpose was to identify published work from between the years 2006 and 2021 on the effects of marine algal species extracts and their bioactive compounds in treating and managing obesity and diabetes. Keywords and phrases included in the search were ‘marine algae and obesity’, ‘marine algae and diabetes’, ‘marine algal bioactive compounds’, ‘marine algae and oxidative stress’, ‘marine algae and lipid accumulation, and ’marine algae on energy intake and adipose mass’. The search yielded 376 articles, which were then screened by reading the titles and abstracts to determine whether they were aligned with the interests of this review. A total of 128 publications were selected based on their abstracts, and then scrutinized by reading through the entire articles; finally, 95 articles on macroalgal species were included in the study. Furthermore, as shown in Figure 5 below, the selected literature was classified according to whether it involved an in vitro study, an in vivo study or a clinical trial of a marine algae compound or extract, and this was used to compile the subsequent results.

## 5. Conclusions

**Figure 6:**
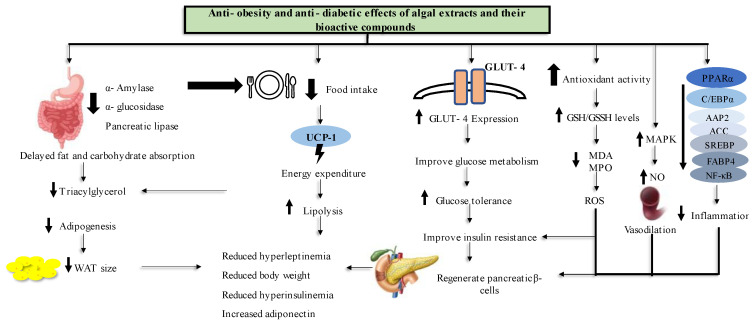
Mechanisms of action for marine algal extracts and their bioactive compounds combating obesity and diabetes. This diagram demonstrates the overall conclusion on the anti-diabetes and anti-obesity effects of marine algae based on this review article.

Obesity and T2D remains an epidemic that seems to be an inevitable challenge for people to overcome. In an attempt to find more natural treatments for these diseases, this review documented evidence for the anti-obesity and anti-T2D effects of marine algal extracts and bioactive compounds and their modes of action from in vitro, in vivo and human study models. Figure 6 shows a conclusive summary of marine algal anti-obesity and anti-diabetes effects as per the literature reviewed in this paper. For in vitro study models, treating the cells with algal extracts and compounds reduced lipid accumulation and genes that are related to adipogenesis. Furthermore, marine algal extracts exhibited potential antioxidant activity. 

Moreover, in the in vivo animal models, it has been demonstrated that marine algae inhibit carbohydrate digestive enzyme activity, which causes a delay in carbohydrate digestion and metabolism. The administration of marine algal extracts and compounds also resulted in a decrease in food intake, which led to increased energy use and fat breakdown. In addition, treating diabetic animals with marine algal products increased GLUT-4 expression, which improved glucose metabolism and reduced insulin resistance. The antioxidant and anti-inflammatory properties of compounds and extracts reversed the pancreatic β- cell damage caused by T2D. Most of the adipogenic genes were under expressed and most of the parameters related to energy utilization were improved after the treatment with marine algae extracts and their bioactive compounds in all experimental settings. 

In clinical trials, the marine algal extracts and compounds reversed the obesity and T2D effects by reducing bodyweight and food intake and improving their plasma biochemical parameters, such as glucose and glucose intolerance, lipoprotein levels, antioxidant defense system and plasma insulin levels. Despite the fact that the in vitro and in vivo literature in this review demonstrated that marine algae have high potential for use as candidates in the development of anti-obesity and anti-diabetes drugs, there were limited human trials to confirm these findings. Furthermore, some compounds and extracts were only studied at the in vitro level, where not all obesity or T2D related parameters were assessed. Moreover, most studies did not assess the toxicity levels of the studied marine algal compounds and extracts to confirm their safe doses or safety for use as a natural medicine.

Overall, fucoxanthin, fucoidan and astaxanthin were widely studied among the primary bioactive compounds, while dieckol, dioxinodehydroeckol (DHE) and pyrogallol-phloroglucionol-6,6′-bieckol (PPB) were widely studied among the secondary bioactive compounds. In terms of algal species, the plants from the *Sargassum* and *Gelidium* genera were studied more compared to other genera and species.

## 6. Recommendations

A huge amount of marine algae goes to waste on the coastlines even though its health benefits have been demonstrated and the demand for naturally derived drugs, nutraceuticals and supplements is very high. Therefore, further dose–response studies are warranted, not only to confirm their most effective doses but also the toxicity levels, especially in human subjects, for the development of marine algal-derived anti-obesity and anti-diabetes medicines, food supplements and other food products.

## Data Availability

Not applicable.
